# Transition and Sustainability of an Online Care Model for People With Parkinson's Disease in Response to the COVID-19 Pandemic

**DOI:** 10.3389/fpubh.2021.772805

**Published:** 2022-02-02

**Authors:** Laura Ketigian, Nicholas Piniella, Kaylie McGivney, Samantha Lui, Austin Dukat, Min-Kyung Jung, Rosemary Gallagher, Adena Leder

**Affiliations:** ^1^Department of Osteopathic Medicine, New York Institute of Technology College of Osteopathic Medicine, Old Westbury, NY, United States; ^2^Department of Physical Therapy, New York Institute of Technology School of Health Professions, Old Westbury, NY, United States

**Keywords:** Parkinson's disease, COVID-19, telehealth, telerehabilitation, Rock Steady Boxing

## Abstract

**Introduction:**

CoronaVirus Disease-2019 (COVID-19) led to social distancing and the need for alternative care models. Telehealth programs for people with Parkinson's (PWP) disease may ensure continuity of care. The goal of this observational survey study was to determine the practicability, satisfaction, and barriers to online programs, their relationship to perceived symptoms, mood, and quality of life, and program sustainability beyond the immediate pandemic.

**Methods:**

In-person Parkinson's programs at New York Institute of Technology College of Osteopathic Medicine transitioned online at the start of the pandemic to include Rock Steady Boxing, Support Groups, and Rock Steady Buddies. A custom online survey sent to 150 participants investigated PD history, symptomatology, level of exercise before and during the pandemic, depression (PHQ-9), quality of life (PDQ-39), and practicability and perceived satisfaction related to these online programs. Descriptive statistics were reported.

**Results:**

Of 69 respondents [mean age of 70.2y (SD 8.4 yrs)], >75% were satisfied with the transition to online programs. Consistent attendance and minimal barriers to programs indicated practicability, with increased adherence to exercise. Of 66 completed PHQ-9s, 22.7% had scores ≥9 (moderate to severe depression); of 61 completed PDQ-39s, scores averaged 21.4; better quality of life than national averages for PWP. Self-perceived physical and mental wellbeing were positively affected.

**Conclusions:**

Results suggest the transition to online programs met the needs of the Parkinson's community in a practicable and sustainable manner during the pandemic. With COVID-19 still prevalent, the current model of blending synchronous online and in-person classes provides a more flexible, sustainable format compared to in-person alone. Institutions may consider including online components to existing programs to promote continuity of care for aging populations as part of best practices.

## Introduction

During the spring of 2020, the onset of severe acute respiratory syndrome coronavirus-2 (SARS-COV-2; COVID-19) led to social distancing, shelter-in-place orders, and in some cases, quarantine (1). These measures, enacted to slow the rate of infection, resulted in negative consequences on health and well-being, particularly in older, vulnerable populations such as people with Parkinson's (PWP) disease (2, 3).

The Parkinson's community at New York Institute of Technology's College of Osteopathic Medicine (NYITCOM) and Health Care Center consists of patients, clients, caregivers, medical personnel, and student volunteers carefully and purposefully built over the past several years. The goal is to ‘fight back against Parkinson's' through instilling bonds of support and friendship amongst people with a common vision (4). In addition to offering medical and rehabilitation services specific for PWP, other in-person programs include support groups (SG) and a Rock Steady Boxing (RSB) program.

With the onset of COVID-19, however, these in-person programs were no longer feasible. Therefore, an alternative model using an online platform for delivery of care was launched as virtual RSB (vRSB), virtual support groups (vSG), and a new online program developed specifically in response to the pandemic, called Rock Steady Buddies (*Buddies*). These online programs aimed to provide continuity of care with an emphasis on health maintenance, management of symptoms, maintaining community bonds, and reducing the effects of social isolation.

### Parkinson's Disease and COVID-19

Parkinson's disease (PD) is a progressive neurodegenerative disorder caused by the loss of dopaminergic neurons in the brain, typically affecting adults aged 60 and older (5). This chronic disease presents with motor symptoms, such as tremor, rigidity, bradykinesia, and postural instability, non-motor symptoms, including anosmia, sleep disturbance, and cognitive deficits, and neuropsychiatric symptoms, notably depression and anxiety, that can adversely affect quality of life (QoL) (6). Older age and comorbid conditions are a leading risk factor of mortality in patients with COVID-19 (7). Although there are no formal reports suggesting PD increases the risk of contracting COVID-19, PWP are generally older and may have multiple comorbidities—including respiratory issues—thus, increasing their risk of serious illness and complications from COVID-19 (3, 7–10). Parkinson's disease is classically managed with an array of pharmacotherapy, and, adjunctively, exercise. In PWP, exercise has shown to improve clinical signs and symptoms and delay underlying disease processes through its neuroprotective and neuroplastic effects (11).

### Exercise in Parkinson's Disease

Exercise is a subcategory of physical activity that is planned, structured, repetitive, purposeful, and intended to improve one or more components of physical fitness (12). Research shows that exercise benefits motor symptoms in PWP and may affect cognition (11). In older adults, exercise has shown to positively affect the acquisition and retention of memory, cognition, and depression (13–15). Despite evidence that exercise can reduce disability and improve QoL in PWP, many remain sedentary (11, 16). Common perceived barriers to exercise in PWP include low outcome expectation, lack of time, and fear of falling (16). These barriers can be addressed through communication with patients, exercise education, and behavioral change interventions targeting self-efficacy–including goal setting, social support, monitoring feedback and progress, and using a patient centered approach (17, 18). Exercise programs for PWP include a wide range of activities and formats, including walking, tai chi, and boxing (11). While exercise in general is beneficial for PWP, aerobic, goal-directed and group exercises may be most effective (19, 20).

Group exercise has been shown to enhance social support and peer bonding, increase enjoyment and compliance, and improve perceived health, more so than individual exercise (19, 20). The reciprocality, accessibility, and reliability of social support and connectedness are important in maintaining and/or initiating behavioral change, such as an exercise routine (21, 22). During the pandemic however, in-person group exercise classes were curtailed. Fortunately, video technology and tele-exercise programs have shown to be an effective mode of providing exercise in the home for people with movement disorders (23) including PD (23, 24).

### Mental Health in Parkinson's Disease

While current interventions for PWP mitigate many of the physical symptoms (5, 25), non-physical symptoms, exacerbated by social isolation and stress from the COVID-19 pandemic, are more difficult to manage. Up to 40% of PWP present with anxiety (26) while 50% present with depression (27); combined with elevated stress levels, social distancing, and lack of usual support systems during the pandemic, feelings of helplessness and isolation may result (28).

Social relationships with family, friends, and support groups—specifically, effective communication and acceptance—are necessary to maintain QoL in PWP (29, 30). Coping with the progression of PD presents challenges that may be relieved through support groups, thus improving QoL, depression, and anxiety in PWP (31). Although in-person support groups are common practice for managing the neuropsychiatric symptoms of PD (32), during the COVID-19 pandemic, barriers such as social distancing and shelter-in-place orders rendered in-person programs unavailable. Similar to tele-exercise, technological advances promote the use of online support groups as an alternative model to access care (32), and thus better serve populations with motor disability and disease.

### Objectives

The purpose of this study was to investigate the practicability, perceived satisfaction, and barriers to participation of online programs developed in response to the COVID-19 pandemic, namely, vRSB, vSG, and *Buddies*. Sustainability of the programs, as the COVID-19 pandemic continues, was also considered. Online group exercise programs may be used to address the limited access to in-person programs resulting from the COVID-19 pandemic while preserving social interactions and providing emotional support. The authors hypothesized that the virtual programs were practicable, with low barriers to participation, and high perceived satisfaction. The current study also investigated self-reported symptomatology, mood and QoL of program participants, with expectations that this PD population had more manageable symptoms, less depression and better QoL in relationship to program participation.

## Methodological Aspects

### Study Design and Approval

This observational survey study was approved by the Institutional Review Board at New York Institute of Technology. Instructions for the survey included the agreement of informed consent upon completion and submission of the survey.

### Participants

**Figure 1:**
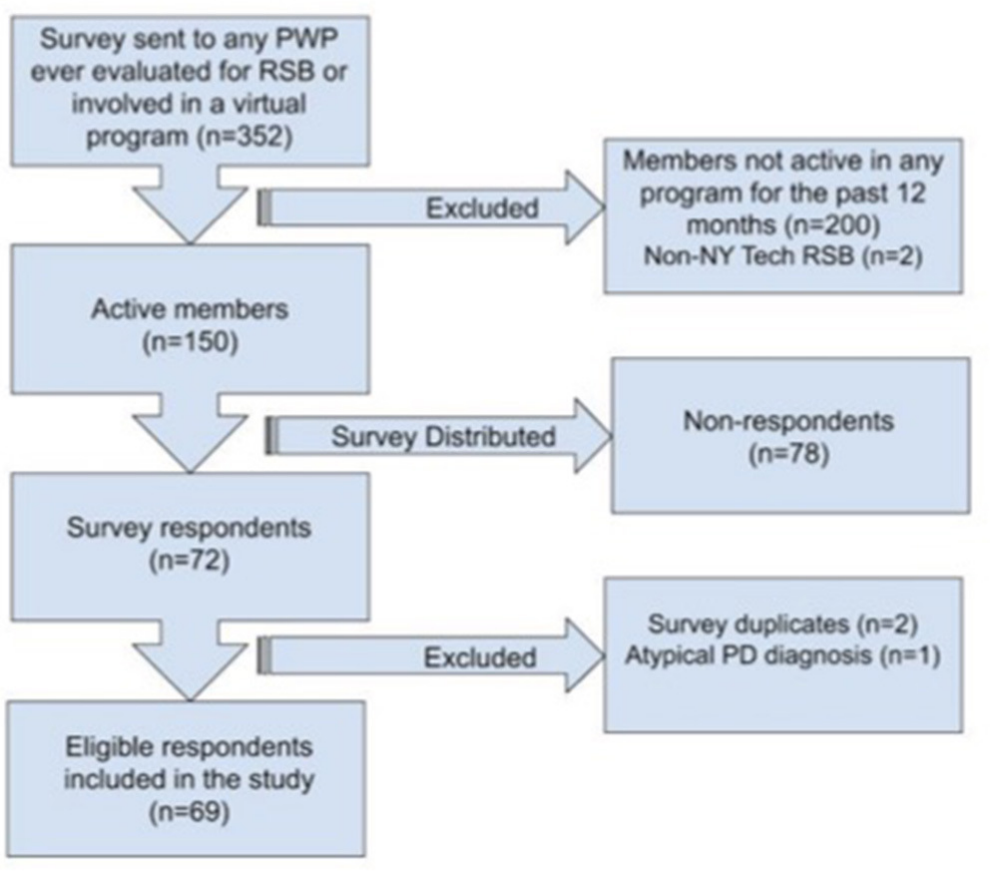
Survey exclusion criteria flowchart.

Participants in online Parkinson's programs were recruited from in-person programs for PWP and telemedicine/telerehabilitation visits at the institutions' Academic Health Care Center. The survey was intended for males and females of any age or PD stage who participated in pRSB classes prior to the pandemic and/or attended any of the three online programs during the pandemic. Participants who responded to the survey, but only attended pRSB before the pandemic, were used as a comparison to PWP who attended online programs during the pandemic for certain measures. People with PD who did not participate in pRSB within the past 12 months, did not participate in at least one online program, had a movement disorder diagnosis other than PD, or were involved in RSB at another institution were not included in the survey analysis (Figure 1). Participation in in-person and/or online programs were adjunctive to pharmacological treatment as determined by participants' neurologists on an individual basis.

### Survey Measurement

A custom online observational survey was developed to gather quantitative and qualitative information regarding motivation, satisfaction, and barriers to online programs. An initial version of the survey was developed by medical and physical therapy students with knowledge of the pathophysiology of PD and experience working directly with boxers in the pRSB program. A movement disorder specialist (AL) and physical therapist with expertise in PD (RG) also contributed to question development. Questions were in closed ended format consisting of leading, matrix, multiple choice, drop down, Likert scale, dichotomous scale, and bipolar rating scale questions. Answer choices ranged from a 4-point Likert scale (strongly disagree, disagree, agree, strongly agree), to multiple choice and short answers. Questions regarding PD symptoms were adapted from the Unified Parkinson's Disease Rating Scale (UPDRS) parts I and II (33).

Five domains were addressed:

1. Demographics and PD history:
2. PD symptomatology since COVID-19: self- assessment of PD progression during the pandemic (^*^Due to social distancing and shelter-in-place orders, PD severity could not be objectively measured)
3. Exercise participation (pre- and post-pandemic)
4. Participation in pRSB: to determine participation in pRSB pre-COVID
5. Participation in online programs (vRSB, vSG, *Buddies*): to determine participation in online programs during COVID.

Collection of information in the aforementioned domains was used to determine practicability — defined by attendance and barriers to program participation — satisfaction, and barriers — defined as obstacles that prevented PWP from participating in the programs.

Due to the high rates of depression in PWP (27) and its negative effect on QoL (6), two validated questionnaires were also used to better understand the mental health and QoL of the participants. The Patient Health Questionnaire-9 (PHQ-9), a screen for depression, consists of 9 DSM-IV criteria (34) scored from 0 (not at all) to 3 (nearly every day). Scores range from 0 (no depression) to 27 (severe depression) (34, 35). The Parkinson's Disease Questionnaire-39 (PDQ-39), is a commonly used health related QoL measure specific to PD. It consists of 39 items covering eight domains: mobility, activities of daily living, emotional well-being, social support, cognition, communication, and bodily discomfort (36). Each item is scored from 0 (best) to 4 (worst). Domain scores range from 0 (high QoL) to 100 (low QoL) (36).

A survey link was emailed 2–3 times a week (14 total) between July 3 and August 8, 2020, using REDCap (Research Electronic Data Capture), a secure, web-based application designed to support data collection and management. The survey was anonymous to reduce evaluator bias and ensure protection of participants' private health information. Approximate survey completion time was 20 min. The survey could be filled out by participants or caregivers if the participant did not have appropriate cognitive reserve to complete the survey independently. The data was analyzed in a descriptive manner.

### Description of Programs

#### In-person Programs: Rock Steady Boxing and Support Groups

Rock Steady Boxing is a non-contact, group-based boxing program that addresses motor and nonmotor symptoms in PWP with the goal to improve coordination, mobility, dexterity, memory, and, ultimately, activities of daily living (11, 20, 37). Prior to the pandemic, the in-person RSB program at NYITCOM consisted of one-hour group exercise classes 3 days/week. Two of the classes were for higher functioning participants and one class for lower functioning participants. Class placement was determined by an evaluation with a physical therapist or movement disorder specialist and included the Unified Parkinson's Disease Rating Scale (UPDRS), the MiniBESTest, and the Montreal Cognitive Assessment (MOCA). Attendance tracked participation. Classes included a range of balance and agility exercises and boxing drills based on RSB principles (38).

The in-person support groups consisted of weekly meetings led by trained medical personnel, as well as a weekly peer-facilitated group of PWP. The initial 8 week program was sponsored by the American Parkinson's Disease Association's *Parkinson's Roadmap for Education and Support Services* (PRESS).

#### Virtual Programs: Rock Steady Boxing, Support Group, and Rock Steady Buddies

**Figure 2:**
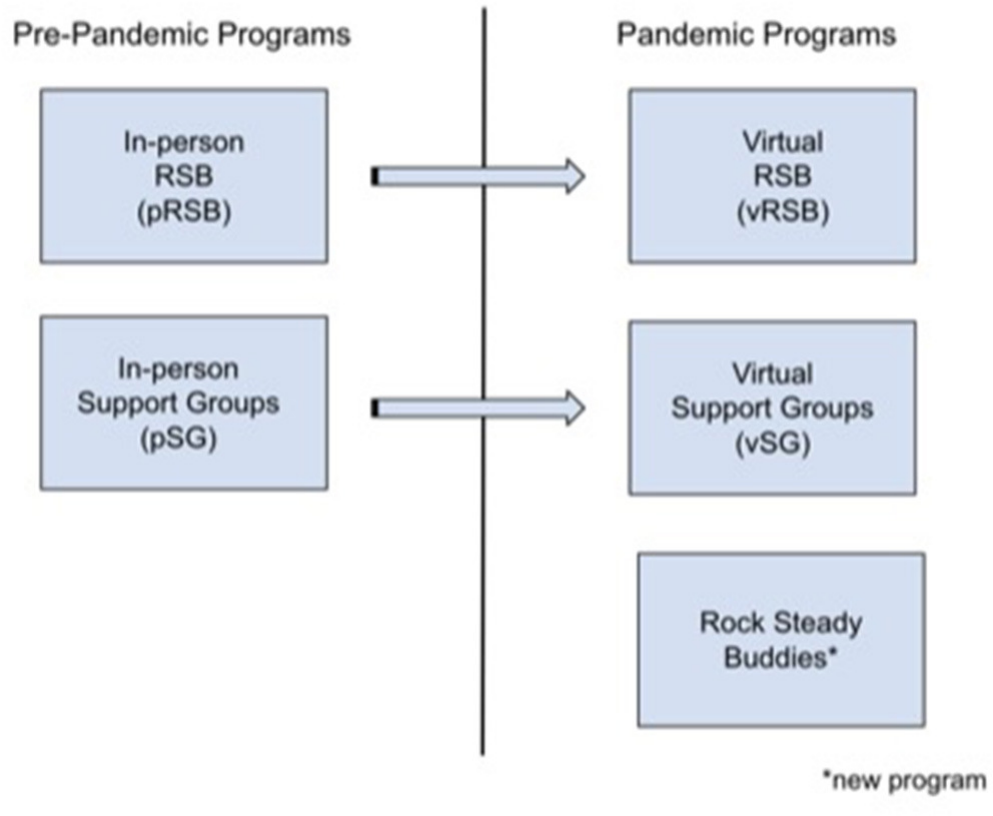
Methodological program flowchart.

The pRSB and pSG were transitioned online at the start of the pandemic to become virtual RSB (vRSB) and virtual SG (vSG) respectively. An additional Rock Steady Buddies program was created to provide individual social support to the PD community (Figure 2). These virtual programs are described below.

##### Virtual Rock Steady Boxing (VRSB)

In-person RSB transitioned to vRSB in March 2020. Zoom video conferencing (San Jose CA, USA) was used for all RSB sessions. The Consensus on Exercising Reporting Template guidelines was used as a basis to describe this program (39). Online sessions followed the same format as in-person sessions with modification of activities depending on available equipment at home. Sessions open 15 min prior to class to allow participant interaction, thus encouraging social connections. Each class begins with a 20-min full-body warm-up including stretching, posture, and balance exercises. Next, boxing drills (35 min)—utilizing punching combinations and footwork—stress coordination and balance and challenge the cardiovascular system. A five-minute cool down completes the class. When applicable, modifications are demonstrated by the trained RSB coach (38). Equipment requirements include appropriate clothing, boxing gloves, and a safe space to exercise. Potential adverse events include fatigue and falls; however, boxers are encouraged to sit when needed and many are accompanied by “cornermen” (spouses/caregivers) as a precaution. During the class, coaches instruct and monitor boxers to ensure appropriate form and class participation; volunteers are also present to ensure all boxers participate in the exercises and offer gentle reminders as needed. Although the boxers in this program were a highly motivated group, boxers could see the coach and other boxers, which may have further increased motivation. It has been shown that social connectedness while exercising may improve exercise adherence (19).

##### Virtual Support Groups (vSG)

In March 2020, NYITCOM also restructured its pSG to vSG with a goal to maintain social bonds and provide timely and accurate information about the pandemic — a practice that, along with continuity of care, may prevent acute clinical worsening and complications (40). Meetings, held one to two times a week *via* Zoom and led by a movement disorders specialist, covered topics such as staying fit and active, and how to safely navigate the community. Webinars and presentations from neurologists, neurosurgeons, physical therapists, and front-line COVID-19 physicians were offered.

##### Rock Steady Buddies (Buddies)

*Buddies* is a medical student-run program created in April 2020 with the aim of providing social connections and support systems for PWP. A secondary goal was to provide early patient exposure for medical students during the pandemic. Medical student volunteers were recruited and randomly matched with interested PWP, with preference for first year medical students to promote early patient exposure. Each pair of *Buddies* self-scheduled a minimum one hour per week to connect *via* phone or video conferencing. Students were provided with “ice-breakers” and conversation starters to use at their discretion such as, “What's something that made you laugh today”, or “What are some of your hobbies”? Oftentimes, PWP would share personal information regarding their PD diagnosis or treatment, however, students were instructed to refrain from answering medical questions or sharing medical or confidential information.

## Results

### Respondent Demographics

The survey was distributed to 150 PWP who participated in in-person and/or online programs from June 2019 to June 2020 (Figure 1). Results are expressed as percentages. Sixty nine respondents with an average age of 70.2 years (SD 8.4, range 47–88) yielded an overall response rate of 46%, which is considered adequate (41). Respondents were majority male (77%), Caucasian (94%) and college educated (74%). Eight percent identified as Hispanic or Latino. Of the 69 respondents, 62 attended pRSB pre-pandemic and 53 participated in at least one online program, 20 attended at least two online programs and seven participated in all three. Overall, 46 respondents attended vRSB, 17 attended vSG, and 24 participated in *Buddies*.

Depression and quality of life were also measured. Of 66 respondents who completed the PHQ-9, 22.7% of the scores were ≥9, which indicates moderate depression. This is below the norms reported in the literature for PWP (42). Of 61 completed PDQ-39's, scores averaged 21.4, which indicates better quality of life compared to normative data (43).

### Self-Reported Symptomatology

**Figure 3:**
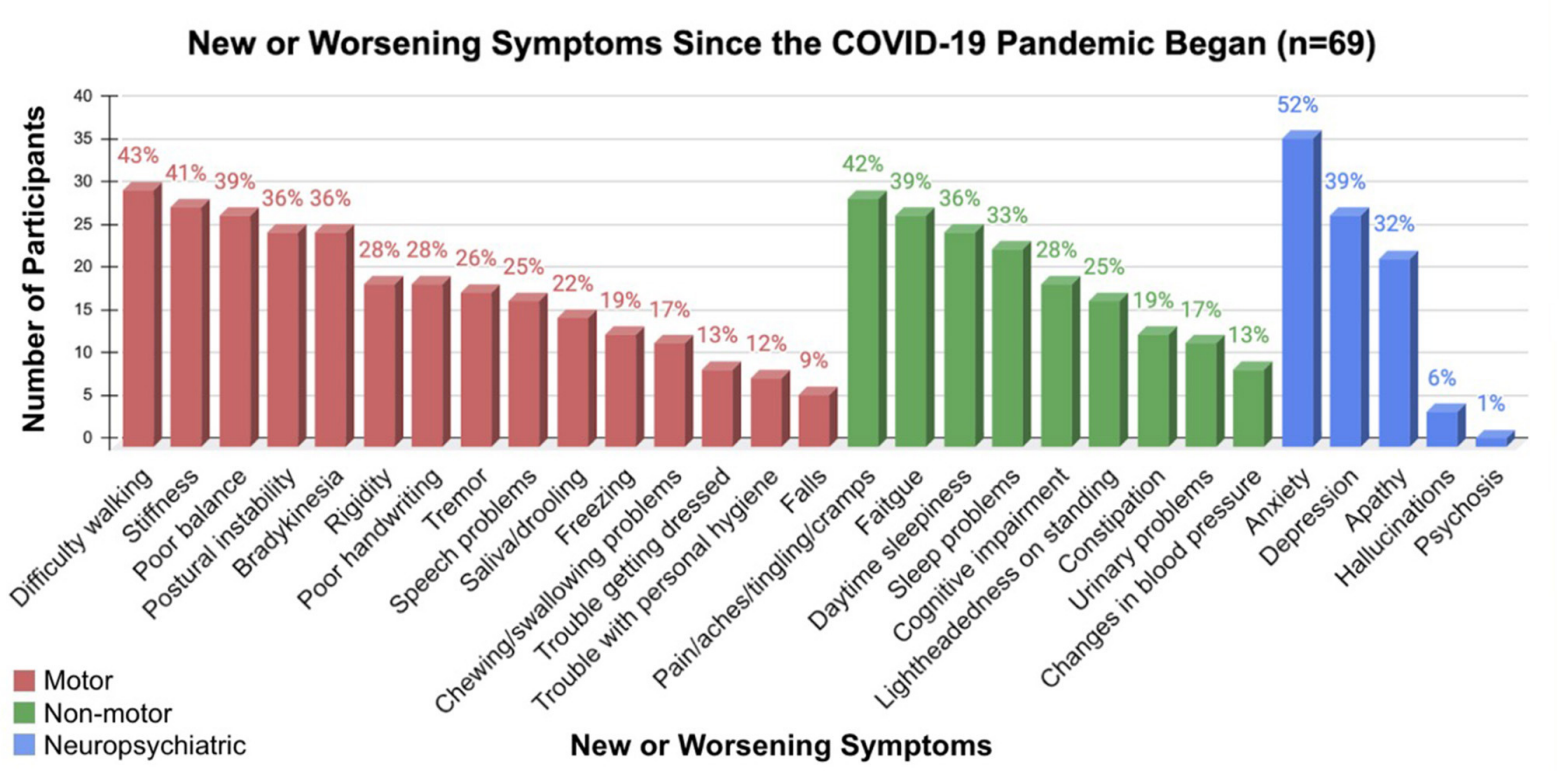
Perceived new or worsening PD symptoms since the start of the COVID-19 pandemic.

Reported symptoms spanned motor, non-motor and neuropsychiatric categories. The most common symptoms were difficulty walking/gait impairment (43%), pain/aches/tingling/cramps (42%), and anxiety (52%) (Figure 3). Almost half reported faster perceived PD progression (41%) and worsening mood (43%). Feelings of loneliness/isolation were high (53%), despite most reporting having a good support system (87%). While 90% of respondents reported at least one new or worsening symptom since the start of the pandemic, participants attending online programs reported an average of 1.3 less new or worsening symptoms than those who only participated in pRSB pre-pandemic.

### Practicability

Prior to the pandemic, an estimated 125 PWP regularly attended at least one pRSB per week. Attendance was not tracked for in person support groups for anonymity purposes. By the end of July 2020, an estimated 75 PWP were taking part in at least one virtual program. Virtual RSB averaged 34 participants for the high functioning class and 21 for the lower functioning class; vSG averaged 30 participants, and Buddies estimated 35 participants. This is in comparison to an estimated average of 20 people per pRSB class, where attendance was capped due to space limitations.

**Figure 4:**
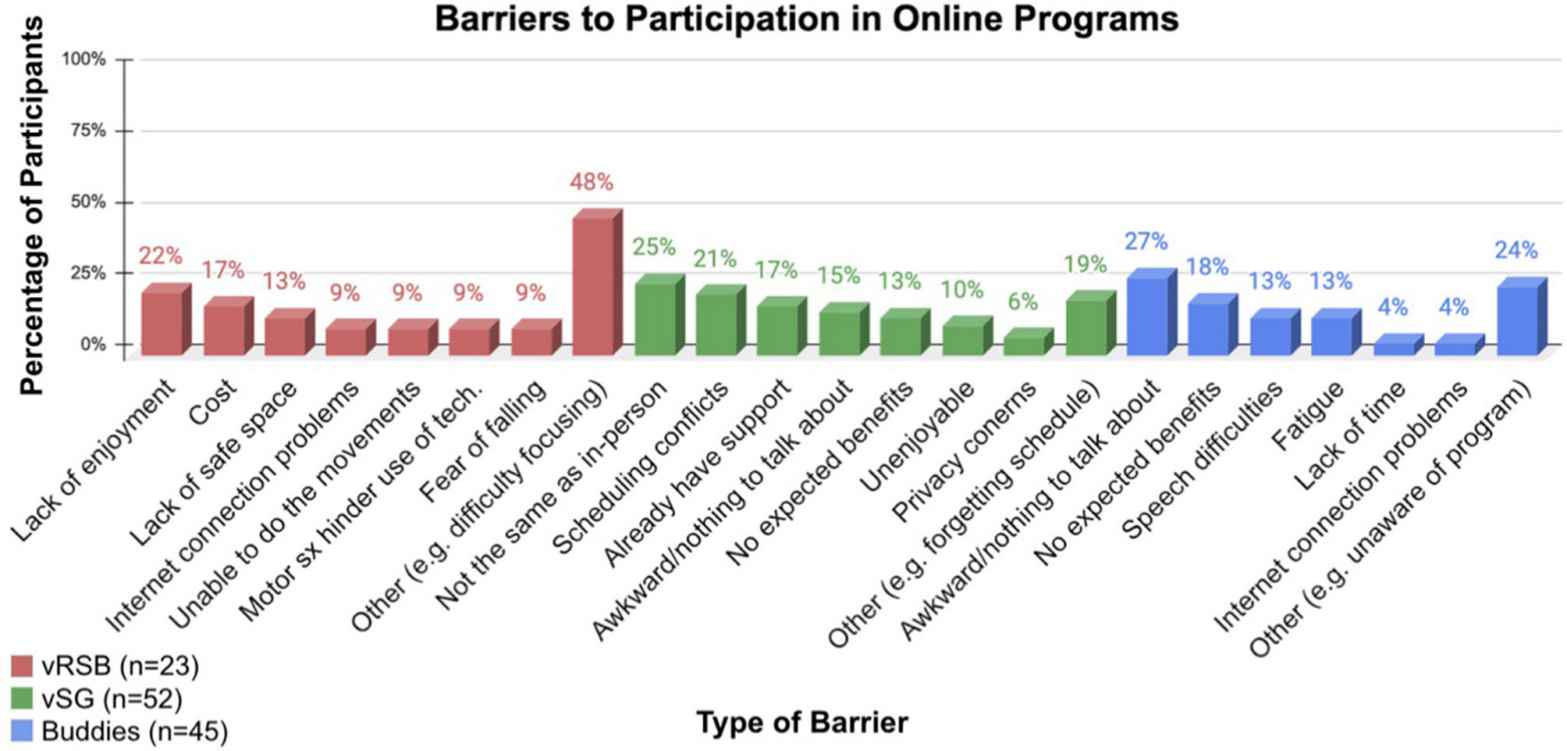
Barriers preventing PWP from participating in our online programs.

Barriers to participation in online programs were few and unique to each program with the most common barrier to vRSB being ‘other' (ex: difficulty focusing) (48%), to vSG, ‘not the same as in-person' (25%) and to Buddies, ‘it would be awkward/I would have nothing to talk about' (27%) (Figure 4).

### Satisfaction

**Figure 5:**
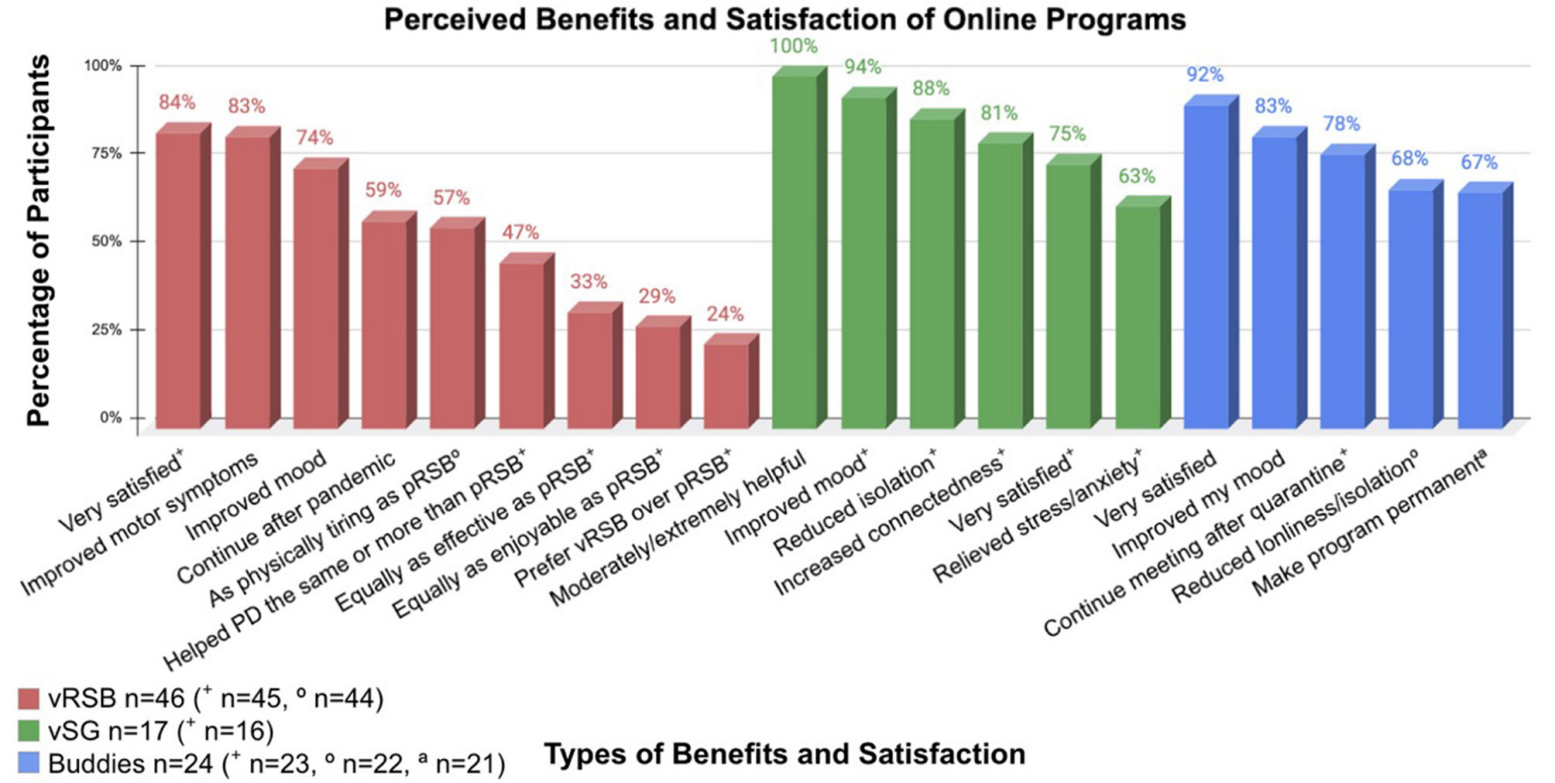
Perceived benefits and satisfaction of the online programs.

Overall, perceived improvements in symptoms and mood, together with the majority of respondents reporting ‘very satisfied' with each program, underscore satisfaction (Figure 5). Here results are presented according to program:

#### vRSB

The majority of vRSB respondents felt the program improved their motor symptoms (83%) and mood (74%), with many reporting they would continue with online classes post pandemic (59%). Satisfaction may also be measured by exercise compliance. Regarding weekly attendance, 62 people (89.9%) reported attending pRSB an average of 1.94 classes per week, whereas 45 people (65.2%) reported attending vRSB an average of 2.67 classes per week. Of the 44 people who reported weekly attendance to *both* pRSB and vRSB, the average increase in classes per week from pRSB to vRSB was 0.55; 59% (n=44) attended vRSB an average of 1.3 classes more per week than pRSB (an average of 1.3 classes).

#### vSG

The majority of vSG participants reported that the program improved mood (94%), reduced isolation (88%), and facilitated connections with friends and their community (81%). Information regarding COVID-19 relieved stress and anxiety in 63% of respondents.

#### Buddies

The majority of participants agreed that meeting with their buddy improved mood (83%) and reduced loneliness (68%). Over 75% reported they would continue meeting with their buddy post-pandemic. Qualitative feedback from respondents included, but was not limited to, the following:

“*100% satisfied. He's a great kid and we learn from each other. Would love to continue.”*

“*The support student I have now is really great. We discuss many topics and enjoy the depth of our conversations. Since my buddy is a first-year med, I hope to be connected with him for a long time.”*

“*My buddies are wonderful, caring, empathetic young people. They have the right personal skills to be great doctors.”*

## Discussion

In response to the COVID-19 pandemic, NYITCOM transitioned to an online platform for exercise (vRSB) and social support (vSG and *Buddies*) to maintain continuity of care for PWP. The results of a custom online observational survey found the online programs to be practicable, with minimal barriers to participation and high rates of satisfaction. Participants also reported perceived benefits relating to depression and QoL. This practicable and comprehensive online plan increases access to care and is currently ongoing in a hybrid format with the programs that have returned to in-person. The long-term plan is to sustain classes in a hybrid manner even after the pandemic has fully subsided.

### Practicability & Barriers

A consistent number of participants and low number of barriers were found in the online classes, thus, attesting to their success and practicability. The literature supports these findings. Telemedicine platforms are a valid and practicable method for assessing PWP (3). When comparing a self-monitored home exercise program to a tele-coach assisted program in PWP, Lai et al. found participants in the tele-coach program had higher attendance (99%) compared to the self-monitored group (35%). More time exercising, especially at a higher intensity, was also found for the tele-coach participants (24), indicating the effectiveness of online exercise programs for PWP.

However, barriers to online programs exist. Older and less educated adults access the internet at a lower rate, 64 and 68% respectively, than younger adults (16). As a demographic, PWP are generally older and experience difficulties with technology that can be further impacted by motor and/or cognitive deficits (44). Despite this, internet access for the vRSB class was a barrier for only 9% of respondents, with little to no internet access problems for vSG or *Buddies*. This may be attributed to the participants in this study living in a suburban area with good internet access, high levels of education and having assistance from family members and/or caregivers. Transportation issues, distance, and weather have also been cited as barriers to exercise (16, 18, 45). Removing the need for travel may have contributed to the facilitation of attendance to online programs.

### Satisfaction, Depression and Quality of Life

Benefits of online programs were underscored by high satisfaction, a perceived positive effect on PD symptoms, and exercise compliance. The findings agree with the literature. Online platforms for health have proven effective for remote diagnosis and treatment (46) including for PWP (3). When compared to in-person visits, telehealth has produced similar satisfaction, patient compliance and QoL (47, 48).

While PHQ-9 and PDQ-39 scores were not obtained prior to COVID-19 for which to compare, the number of PWP reporting depression and poor QoL is relatively low when compared to national averages. This may indicate that these programs and the tight-knit community adequately supported the PWP.

#### The Importance of Physical Activity: VRSB

Exercise is associated with slower progression of disease, and improved function, mobility, and improved QoL in PWP (49). However, quarantining during the pandemic may prevent PWP from an active lifestyle, which may already be impeded by pre-existing conditions (3). Group exercise, such as vRSB, using an online platform may be an alternative for managing motor and non-motor symptoms of PD, even post-pandemic. Through group exercise, social interactions and emotional support can be preserved in older adults, which is associated with improved self-efficacy, reduced risk of depression and all-cause mortality, and greater intrinsic motivation for moderate to vigorous physical activity (21, 48).

While RSB is a long-standing national program, to the authors' knowledge it has never before been employed online (38). The urgency of social distancing due to the COVID-19 pandemic resulted in lack of guidelines on how to deliver the classes in an online format, therefore, this may be one of the first studies to do so. Other institutions—such as the Michael J. Fox Foundation (50)—have begun to offer RSB instruction in an online format. This current study may provide valuable information to those institutions who seek to add an online RSB component to their programming.

#### The Psychological Importance of Social Connections: vSG and Buddies

Human connection is an innate and essential part of life. Diminished contact with others may be associated with lasting negative consequences on physical and mental health (51). High rates of comorbidity in PWP, including depression and anxiety (52), combined with elevated stress, social distancing, and lack of usual support systems during the pandemic created the potential for feelings of helplessness and isolation (28). Quarantining has been associated with negative psychological effects, especially in people with pre-existing poor mental health (28). This may hold true for PWP due to depletion of dopamine. Dopamine in the mesolimbic circuit regulates the processing of emotion and motivation (53). Chronic stress may reduce dopaminergic neurons in the basal ganglia and worsen mental health, especially in PD, where dopamine is already deficient (54). The American Parkinson's Disease Association encourages PWP to manage stress and mental health by establishing and maintaining social connections (4, 55). Virtual support groups and *Buddies* programs addressed these issues.

The aims of vSG were to maintain social connections and to positively influence QoL (25, 31). Other aims included the provision of timely and accurate information about the pandemic (28, 40), thus providing a forum for participants to discuss concerns and fears regarding the pandemic and its potential impact on their health. The findings of the survey show that vSG helped decrease the effects of isolation, foster connections with others and improve mood; these findings reinforce existing literature (28).

NYITCOM's *Buddies* program was developed to provide companionship to offset the effects of isolation in PWP during the pandemic. The secondary aim was to provide osteopathic medical students with early patient exposure. The program was based on existing in-person Buddy programs that focused on student education and interactions with geriatric neurologic patients (56). These programs found benefits for students and patients alike. Participants reported a decreased sense of loneliness and improved mood through participation in the program. Anecdotally, as pairs became closer with one another, they discussed more personal issues regarding difficulties faced due to the COVID-19 pandemic. Some pairs added online games, such as chess, to their weekly conversations. Specific positive feedback from participants regarding their experience with *Buddies* may be interpreted as an indicator of success of the program.

### Sustainability: Online-Programs Today

Once in-person programs resumed in the Spring of 2021, many participants expressed the desire to continue the online programs. With the return of in-person RSB classes, a new “blended” format was developed; an online component has been added to the in-person classes and are held synchronously for participants who find the online format more amenable to their needs. As high as 30% of boxers continue using the online format at this time. Both groups are able to see and interact with each other, thus keeping with the desire to encourage social bonding and support *via* group exercise. This same blended format is planned for our support groups once in-person meetings resume. Rock Steady Buddies has continued to be successful among students and PWP alike. Each semester PD participants are paired with a new Buddy with whom to build new relationships. The program has plans to continue and expand to include older adults from local nursing homes.

Implications for further research include investigating the sustainability of these programs beyond the pandemic. In addition, now that online programs have been found to be effective as well as convenient, together with advances in technology, the use of telehealth and telerehab platforms are likely to become a mainstay in healthcare. This program may serve as a model for other institutions to adopt not only for their population with PD, but other patient populations as well.

### Limitations

Limitations and areas for improvement are addressed here. This was an observational study; therefore, no comparisons were made to pre-COVID-19 program conditions. The PHQ-9 and PDQ-39 are validated tools; however, the survey developed for this study, although based on the UPDRS Parts I and II, was not a validated survey, therefore, future studies may consider utilizing a validated tool. Since the survey was anonymous and completed online, objective measurements of PD severity and participant cognition were not included, therefore, reporting of symptomatology relied on respondents' self-reported scores. Potential response-bias — or more specifically, volunteer or self-selection bias — may have also been present; motivated individuals who enjoyed the programs may have responded to the survey at a higher rate than non-motivated individuals who did not enjoy the programs. Also, while a small subset of participants reported internet access as a barrier to participation, those who responded to the electronically distributed survey inevitably had adequate access to the internet while those with technology challenges may not have participated at all, thus potentially affecting the results. Lastly, results of this study cannot be generalized beyond the demographic group of educated, primarily male, Caucasian, PWP living in a suburban setting.

## Conclusion

While the COVID-19 pandemic compromised standard interventions for PWP, it also created an opportunity to develop innovative approaches for alternative care models. The institution pivoted in-person programs to an online format tailored specifically to address the physical and mental wellbeing of PWP during a global pandemic. The practicability of vRSB, vSG, and *Buddies* programs are underscored by consistent attendance, limited number of barriers to participation, exercise compliance, and the high satisfaction, mood and QoL of participants. This suggests that these programs are beneficial for the physical and mental health of this PD community during the COVID-19 pandemic. Other healthcare and academic institutions may consider implementing similar programs to promote continuity of care for PWP and other aging populations during times of crises, and as part of best practices. Future directions include investigating the sustainability of online programs for PWP and for other patient populations beyond COVID-19.

## Data Availability Statement

The raw data supporting the conclusions of this article will be made available by the authors, without undue reservation.

## Ethics Statement

The studies involving human participants were reviewed and approved by Institutional Review Board at New York Institute of Technology (BHS-1569). The patients/participants provided their written informed consent to participate in this study.

## Author Contributions

All authors provided substantial contributions to conception and design, acquisition of data, or analysis and interpretation of data, drafted the article or revised it critically for important intellectual content, gave final approval of the version of the article to be published, and agreed to be accountable for all aspects of the work in ensuring that questions related to the accuracy or integrity of any part of the work are appropriately investigated and resolved.

## Conflict of Interest

The authors declare that the research was conducted in the absence of any commercial or financial relationships that could be construed as a potential conflict of interest.

## Publisher's Note

All claims expressed in this article are solely those of the authors and do not necessarily represent those of their affiliated organizations, or those of the publisher, the editors and the reviewers. Any product that may be evaluated in this article, or claim that may be made by its manufacturer, is not guaranteed or endorsed by the publisher.

## References

[1] Lewnard JA Lo NC. Scientific and ethical basis for social-distancing interventions against COVID-19. Lancet Infect Dis. (2020) 20:631–3. 10.1016/S1473-3099(20)30190-0 32213329 PMC7118670

[2] Antonini A Leta V Teo J Chaudhuri KR. Outcome of parkinson's disease patients affected by COVID−19. Mov Disord. (2020) 35:905–8. 10.1002/mds.28104 32347572 PMC7267273

[3] Papa SM Brundin P Fung VSC Kang UJ Burn DJ Colosimo C . Impact of the COVID-19 pandemic on Parkinson's disease and movement disorders. Mov Disord Off J Mov Disord Soc. (2020) 35:711–5. 10.1002/mds.28067 32250460 PMC7996401

[4] Online Parkinson's disease resources| APDA (2021). Available online at: https://www.apdaparkinson.org/article/online-parkinsons-disease-resources/

[5] DeMaagd G Philip A. Parkinson's disease and its management: part 1: disease entity, risk factors, pathophysiology, clinical presentation, and diagnosis. P T Peer-Rev J Formul Manag. (2015) 40:504–32. 26236139 PMC4517533

[6] Martínez-Martín P. An introduction to the concept of “quality of life in Parkinson's disease.” J Neurol. (1998) 245:S2–6. 10.1007/PL00007733 9617714

[7] Kurtis M Mir P. UPDATE: treating COVID-19 in PD and other Movement Disorders: A review of drug interactions. (2020). Available online at: https://www.movementdisorders.org/MDS/Education/Workshops-Conferences/MDS-Webinars/Live/DRUG20WEB2.htm

[8] Csoti I Jost WH Reichmann H. Parkinson's disease between internal medicine and neurology. J Neural Transm. (2016) 123:3–17. 10.1007/s00702-015-1443-z 26298728 PMC4713462

[9] Fasano A Antonini A Katzenschlager R Krack P Odin P Evans AH . Management of advanced therapies in Parkinson's disease patients in times of humanitarian crisis: The COVID−19 experience. Mov Disord Clin Pract. (2020) 7:361–72. 10.1002/mdc3.12965 32373652 PMC7197306

[10] Parkinson's UK,. Parkinson's Coronavirus. Your Questions Answered. (2020, May). Available online at: Www.Parkinsons.Org.Uk; https://www.parkinsons.org.uk/sites/default/files/2020-05/Parkinson%27s%20and%20Coronavirus%20-%20your%20questions%20answered.pdf

[11] Ellis T Rochester L. Mobilizing Parkinson's Disease: The future of exercise. Brundin P Langston JW Bloem BR editors. J Park Dis. (2018) 8:S95–100. 10.3233/JPD-181489 30584167 PMC6311359

[12] What, does “physical activity” mean? Available online at: https://www.euro.who.int/en/health-topics/disease-prevention/physical-activity/news/news/2011/02/being-physically-active-helps-prevent-cancer/what-does-physical-activity-mean

[13] Creer DJ Romberg C Saksida LM van Praag H Bussey TJ. Running enhances spatial pattern separation in mice. Proc Natl Acad Sci. (2010) 107:2367–72. 10.1073/pnas.0911725107 20133882 PMC2836679

[14] Dunn AL Trivedi MH Kampert JB Clark CG Chambliss HO. Exercise treatment for depression. Am J Prev Med. (2005). 28:1–8. 10.1016/j.amepre.2004.09.003 15626549

[15] Izquierdo I Bevilaqua LRM Rossato JI Bonini JS Silva WCD Medina JH . The connection between the hippocampal and the striatal memory systems of the brain: A review of recent findings. Neurotox Res. (2006) 10:113–21. 10.1007/BF03033240 17062373

[16] Ellis T Boudreau JK DeAngelis TR Brown LE Cavanaugh JT Earhart GM . Barriers to exercise in people with parkinson disease. Phys Ther. (2013) 93:628–36. 10.2522/ptj.20120279 23288910 PMC3641403

[17] Ene H McRae C Schenkman M. Attitudes toward exercise following participation in an exercise intervention study. J Neurol Phys Ther. (2011) 35:34–40. 10.1097/NPT.0b013e31820cb917 21475082

[18] Quinn L Macpherson C Long K Shah H. Promoting physical activity *via* telehealth in people with parkinson disease: the path forward after the COVID-19 pandemic? Phys Ther. (2020) 100:1730–6. 10.1093/ptj/pzaa128 32734298 PMC7454884

[19] Kanamori S Takamiya T Inoue S Kai Y Kawachi I Kondo K. Exercising alone versus with others and associations with subjective health status in older Japanese: The JAGES Cohort Study. Sci Rep. (2016) 6:39151. 10.1038/srep39151 27974855 PMC5156899

[20] Petzinger GM Fisher BE McEwen S Beeler JA Walsh JP Jakowec MW. Exercise-enhanced neuroplasticity targeting motor and cognitive circuitry in Parkinson's disease. Lancet Neurol. (2013) 12:716–26. 10.1016/S1474-4422(13)70123-6 23769598 PMC3690528

[21] Lindsay Smith G Banting L Eime R O'Sullivan G van Uffelen JGZ. The association between social support and physical activity in older adults: a systematic review. Int J Behav Nutr Phys Act. (2017) 14:56. 10.1186/s12966-017-0509-8 28449673 PMC5408452

[22] Williams DM. Exercise, Affect, and Adherence: An Integrated Model and a Case for Self-Paced Exercise. J Sport Exerc Psychol. (2008). 30:471–96. 10.1123/jsep.30.5.471 18971508 PMC4222174

[23] Chen JJ Cooper DM Haddad F Sladkey A Nussbaum E Radom-Aizik S. Tele-exercise as a promising tool to promote exercise in children with cystic fibrosis. Front Public Health. (2018). 6:269. 10.3389/fpubh.2018.00269 30324099 PMC6172297

[24] Lai B Bond K Kim Y Barstow B Jovanov E Bickel CS. Exploring the uptake and implementation of tele-monitored home-exercise programmes in adults with Parkinson's disease: A mixed-methods pilot study. J Telemed Telecare. (2020). 26:53–63. 10.1177/1357633X18794315 30134777

[25] Subramanian I. Virtual Parkinson's disease support groups in the COVID−19 era: social connection in the time of social distancing. Mov Disord Clin Pract. (2020). 7:739–40. 10.1002/mdc3.12994 32775536 PMC7300459

[26] Chen P-H Cheng S-J. Depression in Parkinson disease: current understanding and treatment. Int J Gerontol. (2008). 2:172–82. 10.1016/S1873-9598(09)70006-7

[27] McDonald WM Richard IH DeLong MR. Prevalence, etiology, and treatment of depression in Parkinson's disease. Biol Psychiatry. (2003). 54:363–75. 10.1016/S0006-3223(03)00530-4 12893111

[28] Brooks SK Webster RK Smith LE Woodland L Wessely S Greenberg N . The psychological impact of quarantine and how to reduce it: rapid review of the evidence. The Lancet. (2020). 395:912–20. 10.1016/S0140-6736(20)30460-8 32112714 PMC7158942

[29] Ghorbani Saeedian R Nagyova I Krokavcova M Skorvanek M Rosenberger J Gdovinova Z . The role of social support in anxiety and depression among Parkinson's disease patients. Disabil Rehabil. (2014). 36:2044–9. 10.3109/09638288.2014.886727 24533876

[30] Takahashi K Kamide N Suzuki M Fukuda M. Quality of life in people with Parkinson's disease: the relevance of social relationships and communication. J Phys Ther Sci. (2016). 28:541–6. 10.1589/jpts.28.541 27065542 PMC4793007

[31] Artigas NR Striebel VLW Hilbig A Rieder CR de M. Evaluation of quality of life and psychological aspects of Parkinson's disease patients who participate in a support group. Dement Neuropsychol. (2015). 9:295–300. 10.1590/1980-57642015dn93000013 29213975 PMC5619372

[32] Lieberman MA Winzelberg A Golant M Wakahiro M Diminno M Aminoff M . Online support groups for Parkinson's patients: a pilot study of effectiveness. Soc Work Health Care. (2006). 42:23–38. 10.1300/J010v42n02_02 16390834

[33] Perlmutter JS. Assessment of Parkinson disease manifestations. Curr Protoc Neurosci. (2009). 49:10.1.1-10.1.14. 10.1002/0471142301.ns1001s49 19802812 PMC2897716

[34] Kroenke K Spitzer RL Williams JBW. The PHQ-9: Validity of a brief depression severity measure. J Gen Intern Med. (2001). 16:606–13. 10.1046/j.1525-1497.2001.016009606.x 11556941 PMC1495268

[35] PHQ-9 Depression Test Questionnaire (2020). Available from: https://patient.info/doctor/patient-health-questionnaire-phq-9

[36] Nojomi M Mostafavian Z Shahidi GA Jenkinson C. Quality of life in patients with Parkinson's disease: Translation and psychometric evaluation of the Iranian version of PDQ-39. J Res Med Sci Off J Isfahan Univ Med Sci. (2010) 15:63–9. 21526061 PMC3082793

[37] Combs SA Diehl MD Staples WH Conn L Davis K Lewis N . Boxing training for patients with parkinson disease: a case series. Phys Ther. (2011). 91:132–42. 10.2522/ptj.20100142 21088118

[38] Rock Steady. (2020). Available from: https://www.rocksteadyboxing.org/

[39] Slade SC Dionne CE Underwood M Buchbinder R. Consensus on exercise reporting template (CERT): explanation and elaboration statement. Br J Sports Med. (2016). 50:1428–37. 10.1136/bjsports-2016-096651 27707738

[40] Schirinzi T Cerroni R Di Lazzaro G Liguori C Scalise S Bovenzi R . Self-reported needs of patients with Parkinson's disease during COVID-19 emergency in Italy. Neurol Sci. (2020). 41:1373–5. 10.1007/s10072-020-04442-1 32363506 PMC7196180

[41] Story DA Tait AR. Survey research. Anesthesiology. (2019) 130:192–202. 10.1097/ALN.0000000000002436 30688782

[42] Chagas MHN Tumas V Rodrigues GR Machado-de-Sousa JP Filho AS Hallak JEC . Validation and internal consistency of Patient Health Questionnaire-9 for major depression in Parkinson's disease. Age Ageing. (2013). 42:645–9. 10.1093/ageing/aft065 23761457

[43] Hagell P Nygren C. The 39 item Parkinson's disease questionnaire (PDQ-39) revisited: implications for evidence based medicine. J Neurol Neurosurg Amp Psychiatry. (2007). 78:1191–8. 10.1136/jnnp.2006.111161 17442762 PMC2117601

[44] Schneider RB Biglan KM. The promise of telemedicine for chronic neurological disorders: the example of Parkinson's disease. Lancet Neurol. (2017). 16:541–51. 10.1016/S1474-4422(17)30167-9 28566190

[45] Bland KA Bigaran A Campbell KL Trevaskis M Zopf EM. Exercising in isolation? The role of telehealth in exercise oncology during the COVID-19 pandemic and beyond. Phys Ther. (2020). 100:1713–6. 10.1093/ptj/pzaa141 32737965 PMC7454921

[46] Chaet D Clearfield R Sabin JE Skimming K. Ethical practice in telehealth and telemedicine. J Gen Intern Med. (2017). 32:1136–40. 10.1007/s11606-017-4082-2 28653233 PMC5602756

[47] Sekimoto S Oyama G Hatano T Sasaki F Nakamura R Jo T . A randomized crossover pilot study of telemedicine delivered *via* iPads in Parkinson's disease. Park Dis. (2019) 2019:1–7. 10.1155/2019/9403295 30723541 PMC6339724

[48] Kahn E Ramsey L Brownson R Heath G Howze E Powell K . The effectiveness of interventions to increase physical activityA systematic review1 and 2. Am J Prev Med. (2002). 22:73–107. 10.1016/S0749-3797(02)00434-8 11985936

[49] Oguh O Eisenstein A Kwasny M Simuni T. Back to the basics: Regular exercise matters in Parkinson's disease: Results from the National Parkinson Foundation QII Registry study. Parkinsonism Relat Disord. (2014). 20:1221–5. 10.1016/j.parkreldis.2014.09.008 25258329

[50] Kristy Rose Follmar CPT. Rock steady boxing offers new video series to keep people active, safe and connected | Parkinson's disease. (2021). Available online at: https://www.michaeljfox.org/news/rock-steady-boxing-offers-new-video-series-keep-people-active-safe-and-connected

[51] Sikali K. The dangers of social distancing: How COVID-19 can reshape our social experience. J Community Psychol. (2020). jcop.22430. 10.1002/jcop.22430 32880991 PMC7461541

[52] Timmer MHM van Beek MHCT Bloem BR Esselink RAJ. What a neurologist should know about depression in Parkinson's disease. Pract Neurol. (2017). 17:359–68. 10.1136/practneurol-2017-001650 28739866

[53] Douma EH de Kloet ER. Stress-induced plasticity and functioning of ventral tegmental dopamine neurons. Neurosci Biobehav Rev. (2020). 108:48–77. 10.1016/j.neubiorev.2019.10.015 31666179

[54] Sugama S Sekiyama K Kodama T Takamatsu Y Takenouchi T Hashimoto M . Chronic restraint stress triggers dopaminergic and noradrenergic neurodegeneration: Possible role of chronic stress in the onset of Parkinson's disease. Brain Behav Immun. (2016). 51:39–46. 10.1016/j.bbi.2015.08.015 26291405 PMC4849407

[55] Simpson J Haines K Lekwuwa G Wardle J Crawford T. Social support and psychological outcome in people with Parkinson's disease: Evidence for a specific pattern of associations. Br J Clin Psychol. (2006). 45:585–90. 10.1348/014466506X96490 17076966

[56] Cumberland DM Sawning S Church-Nally M Shaw MA Branch E LaFaver K. Experiential learning: transforming theory into practice through the parkinson's disease buddy program. Teach Learn Med. (2019). 31:453–65. 10.1080/10401334.2019.1580583 30860904

